# The significance of *FMR1* CGG repeats in Chinese women with premature ovarian insufficiency and diminished ovarian reserve

**DOI:** 10.1186/s12958-020-00645-5

**Published:** 2020-08-12

**Authors:** Ruiyi Tang, Qi Yu

**Affiliations:** Department of Obstetrics and Gynecology, Peking Union Medical College Hospital, Peking Union Medical College, Chinese Academy of Medical Science, No 1 Shuaifuyuan, Wangfujing, Beijing, 100730 DongCheng District China

**Keywords:** Premature ovarian insufficiency, Diminished ovarian reserve, Premutations, *FMR1* alleles, Chinese population

## Abstract

**Background:**

Previous studies have shown that there is an association between *FMR1* CGG repeats and ovarian dysfunction. The aim of this study is to assess the association between the number of CGG repeats in *FMR1* in Chinese patients with premature ovarian insufficiency (POI) and diminished ovarian reserve (DOR).

**Methods:**

This is a cross-sectional, case-control study, which enrolled 124 patients with POI, 57 patients with DOR and 111 normal menopausal controls. The demographic details along with other clinical data were recorded. The *FMR1* CGG repeats were analyzed by polymerase chain reaction and microfluidic capillary electrophoresis.

**Results:**

We could detect two premutation carriers in the POI group (1.6%) and one in the control group (0.9%). No premutation carriers were identified in the DOR group. The frequency of *FMR1* premutations was not different between POI or DOR and controls. The most common CGG repeat was 29 and 30, and the repeat length for allele 2 had a secondary peak around 36–39 repeats. The CGG repeats were divided into groups of five consecutive values, and the distribution of allele 1 in the POI group was different from that in the control group (*P* < 0.001). No statistically significant differences were found for allele 1 between DOR group vs. controls, and for allele 2 between three groups (*P* > 0.05).

**Conclusions:**

The study shows that the frequency of *FMR1* premutations is relatively low (1.6%) in Chinese women with POI. The distribution of allele 1 CGG repeat in patients with POI showed difference from that in healthy women.

## Introduction

The fragile X mental retardation 1 (*FMR1*) gene, located at Xq27.3, is an X-linked gene, carrying CGG repeats in the 5′-untranslated region. Based on the criteria stated by the American College of Medical Genetics and Genomics (ACMG), *FMR1* CGG repeats can be classified as normal (< 45 repeats), intermediate (45–54 repeats), premutation (55–199 repeats), and full mutation (> 200 repeats) [1].

The ovarian reserve indicates a woman’s reproductive potential, which is a function of the number and quality of oocytes [2]. Premature ovarian insufficiency (POI) is a condition that accentuates the extreme spectrum of impaired ovarian function. It is characterized by failure of ovarian function in women less than 40 years of age [3]. The decline of ovarian reserve is a continuous, gradual process and the concept of decreased or diminished ovarian reserve (DOR) has been explored in few reports earlier [4]. DOR is not an overt phenotype and was once described as ‘occult POI’ [5]. Compared with women of similar age, women with DOR commonly have regular menses but they also have reduced number of ovarian follicles and reduced fecundity [2]. It is thought that DOR may or may not progress into POI eventually (depending on whether amenorrhea occurs before the age of 40 years).

Numerous studies have examined the association between *FMR1* CGG repeats and ovarian dysfunction [6–9]. In western countries, premutation of *FMR1* are reportedly correlated with POI in women [7–9], which is now referred to as fragile-X-associated primary ovarian insufficiency (FXPOI). It has been estimated that around 11–14% of familial and 2–6% of sporadic POI cases are associated with *FMR1* premutations [3]. Association between normal or intermediate range *FMR1* alleles and a reproductive risk has also been explored [10, 11]. Multiple association studies of *FMR1* alleles, i.e. CGG repeats > 36 [12], 41–58 [13], 45–54 [9], and 35–54 [14] have been reported to be associated with POI. However, the distribution of *FMR1* CGG repeats also varies with ethnicity [15]. Fewer patients with POI from Asia carried the *FMR1* premutations [16].

The pathogenic role of *FMR1* premutations in Chinese women is controversial. Three earlier studies have shown very low prevalence of premutation carriers in Chinese women with POI (< 1%) [17–19], which is lower than studies from western countries [3]. Therefore, *FMR1* premutation may not be a common explanation for POI in Chinese women.

With regard to the relationship between DOR and *FMR1*, the results are inconsistent: Women with DOR might be at a risk of carrying alleles in the premutation range [5, 20]. Some studies found an association between DOR and normal/intermediate CGG repeats, including < 26 [21], < 28 [22], 35–54 [23], 45–54 [9, 24], or > 40 [5] and have reported a negative effect on ovarian ageing. By contrast, some studies reported that ovarian reserve was not affected by CGG repeats [23–27]. The role of *FMR1* in Chinese women with DOR has not been extensively investigated.

The present study assessed the distribution of the *FMR1* CGG repeat numbers in Chinese women with POI, DOR and compare it to the control group of natural menopausal women. The primary aim of this study is to establish whether CGG repeats are different in the Chinese population of POI and DOR within normal women. The association between the numbers of CGG repeats and endocrine profiles of these patients was also evaluated.

## Methods

This study protocol was approved by the Institutional Review Board (IRB) of Peking Union Medical College Hospital (PUMCH) (No. JS-1604).

### Study participants

One hundred twenty-four women with POI and 57 women with DOR were included in this cross-sectional case-control study. All women were clinically diagnosed with POI or DOR between May 2018 and December 2019 in the Department of Gynecological Endocrinology and Reproductive Medicine of PUMCH. The inclusion criteria for POI group were: amenorrhea (≥ 6 months) before the age of 40 years and elevated serum follicle-stimulating hormone (FSH) levels (> 25 IU/L). The inclusion criteria for DOR group: having regular or irregular menstrual cycles, FSH > 10 IU/L on days 2–4 of menstrual cycle and/or anti-mullerian hormone (AMH) levels ≤1.1 ng/ml on any day [28] before the age of 40 years. All women had normal karyotype and had no family history of fragile X syndrome (FXS). Women who had a history of autoimmune disease, pelvic surgery or chemo/radiotherapy were excluded.

After obtaining written informed consent, blood samples were obtained from each participant for both phenotypic and genotypic analysis. The blood samples from women with regular menstruation were collected during the early follicular phase for measurements of sex hormones. There was no restriction on the day of blood collection for women with amenorrhea. The serum estradiol (E_2_) and FSH were measured at PUMCH Clinical laboratory using a chemiluminescence immunoassay (Roche®, Switzerland) and AMH was measured using an electro-chemiluminescent assay (Roche®, Switzerland). A detailed clinical questionnaire was filled for each subject, which included details like menstrual and reproductive history along with personal and family history.

The control group included 111 post-menopausal women. The comparison data is from the PUMCH Aging Longitudinal Cohort of Midlife Women (PALM), a prospective, open-cohort, which involved with a community-based longitudinal study, aiming to investigate ovarian ageing in midlife women in China [29]. The participants were middle-aged female residents of the TieEr community, Xicheng District, Beijing, China. The inclusion criteria were women who had undergone natural menopause after the age of 40 years, no history of severe systemic diseases, no use of hormonal medications in the previous 3 months, no history of any gynecological endocrine diseases, and not pregnant or lactating in the previous 6 months. An additional blood sample was obtained from each participant for *FMR1* analysis during the follow up in 2018.

### FMR1 assay measures

Genomic DNA was extracted from peripheral blood leukocytes of all participants using standard procedures using commercial DNA extraction kits (Tiangen, Beijing, China). The *FMR1* repeat region was amplified by polymerase chain reaction (PCR) using the FragilEase PCR reagent kit (PerkinElmer, USA) following manufacturer’s protocol. Following primers were used: forward (TCAGGCGCTCAGCTCCGTTTCGGTTTCA), reverse (FAM-AAGCGCCATTGGAGCCCCGCACTTCC). Two female reference DNA samples (30/80 repeats and 20/200 repeats) were obtained from the Coriell Institute for Medical Research (Camden, US) to evaluate the analytical performance of the assay. The reference samples were concurrently amplified. All PCR products were purified using the PureLink PCR Micro Kit (Invitrogen) prior to electrophoresis. And a microfluidic capillary electrophoresis instrument (LabChip® MultiDX) was used to estimate allele sizing. Calculation of the CGG repeat lengths was performed using FraXsoft analysis software (PerkinElmer, USA) by utilizing base pair size data. An analytical and clinical validation of this kit has been performed previously, which proved that the CGG repeats obtained using this assay are highly concordant with those obtained using the conventional reference method (PCR + Southern blot) for 112 archived samples, including 25 samples with a full mutation (the largest allele repeat was 1380 repeats) [30]. The intra-assay (coefficient of variation< 2.5%) and inter-assay imprecision was within 1 repeat.

### Statistical analysis

The *FMR1* analysis in women provides two numbers of the CGG repeat length on two X chromosomes. Consistent with the methodologies used in earlier studies [15, 31], the allele with the smaller number of CGG repeats was termed “allele 1”, and the allele with the larger number of CGG repeats was termed “allele 2”. Continuous variables with normal distributions were expressed as mean ± standard deviation (SD). Categorical variables are presented as numbers (percentages). Discrete categories for CGG repeat categories were presented per 5 repeat lengths. The categorical distributions of allele 1 and allele 2 CGG repeat lengths were compared among different groups. Comparisons of categorical allele variables were made using the non-parametric Mann-Whitney test. Association analysis of continuous variables was used t-testing (two groups) or ANOVA (three or more groups).

Since some studies have shown 25–34 repeats as “normal” [21], a comparison of the proportion of alleles 1 and 2 was made with < 25, 25–34, and > 34 repeats between the POI or DOR group and the controls. A study which included primarily the Caucasian race (76%) and 11% of Asian race have compared the infertile women with DOR with the general female population, and found that the DOR group was more likely to have 35–44 CGG repeats (14.5% vs. 3.9%) [23]. Therefore, comparisons were also made between different groups with respect to 35–44 CGG repeats. Previous reports also showed the onset of amenorrhea occurred significantly earlier in the patients with > 38 CGG repeats [12]. The comparison of menopausal age between women having allele 2 with ≤38 repeats and > 38 repeats was done using Fisher’s exact test with alpha < 0.05.

All analyses were performed using SPSS software (version 24.0 for the OS X system; IBM). All the tests were two-sided with 0.05 significance level.

## Results

### Baseline characteristics

We estimated the CGG repeat numbers in the *FMR1* gene of 124 patients with POI, 57 women with DOR, and 111 controls. Table 1 shows the characteristics of the three groups. Eight of the 124 women with POI were primary amenorrhea, whereas 116 women were secondary amenorrhea, and the mean ± SD of menopausal age was 28.4 ± 7.1 years. In the DOR group, more than half the women (50.9%) had FSH values between 10 and 15 IU/L, 29.8% had FSH between 15.1 and 20.0 IU/L, and 12.3% had FSH > 20 IU/L. About 7.0% of women with DOR had FSH values lower than 10 IU/L, but had AMH values lower than 1.1 ng/ml. Forty-one (71.9%) women had both increased FSH and reduced AMH values.

**Table 1:** Characteristics of patients with POI, DOR and control women

Parameter	POI	DOR	Control^**b**^	P_**1**_^a^	P_**2**_^a^	P_**3**_^a^
**N**	124	57	111			
**Enrolled age, yrs, mean ± SD**	31.3 ± 6.5	32.5 ± 3.8	48.0 ± 6.1	**< 0.001**	**< 0.001**	0.149
**Age at menarche, yrs, mean ± SD**	13.4 ± 1.6	13.2 ± 1.2				0.395
**Age at menopause, yrs, mean ± SD**	28.4 ± 7.1	NA	50.7 ± 3.0	**< 0.001**		
**BMI (kg/m**^**2**^**), mean ± SD**	22.1 ± 3.8	21.6 ± 3.5	25.0 ± 3.2	**< 0.001**	**< 0.001**	0.351
**Currently Smoking, No. (%)**	3 (2.7)	1 (1.8)	2 (1.8)	0.307	0.565	0.593
**FSH (IU/l), mean ± SD**	81.7 ± 35.8	15.0 ± 4.8	30.5 ± 30.8	**< 0.001**	**< 0.001**	**< 0.001**
**Estradiol (pg/ml), mean ± SD**	33.3 ± 38.4	46.4 ± 27.6	87.7 ± 125.1	**< 0.001**	**0.001**	**0.027**
**AMH (ng/ml), mean ± SD**	0.06 ± 0.18	0.80 ± 0.76				**< 0.001**
**FMR1 Allele1, mean ± SD**	29.8 ± 2.1	29.3 ± 3.5	29.9 ± 3.8	0.694	0.281	0.330
**Median [IQR]**	29 [29,30]	30 [29,31]	30 [29,31]			
**FMR1 Allele2, mean ± SD**	33.4 ± 7.7	32.2 ± 4.1	33.2 ± 5.0	0.847	0.202	0.290
**Median [IQR]**	30 [30,36]	31 [30, 34.5]	31 [30,37]			

^a^P_1_ indicated POI versus controls, P_2_ indicated DOR versus controls, and P_3_ indicated POI versus DOR

^**b**^ the characteristics presented in the Table were results at baseline assessment of the women in the control group

Abbreviation: *POI* Premature ovarian insufficiency, *DOR* Diminished ovarian reserve, *FSH* Follicle-stimulating hormone, *AMH* Anti-mullerian hormone, *SD* Standard deviation, *BMI* Body mass index, *IQR* Interquartile range, *NA* Not applicable

### FMR1 CGG repeat distribution

**Fig. 1:**
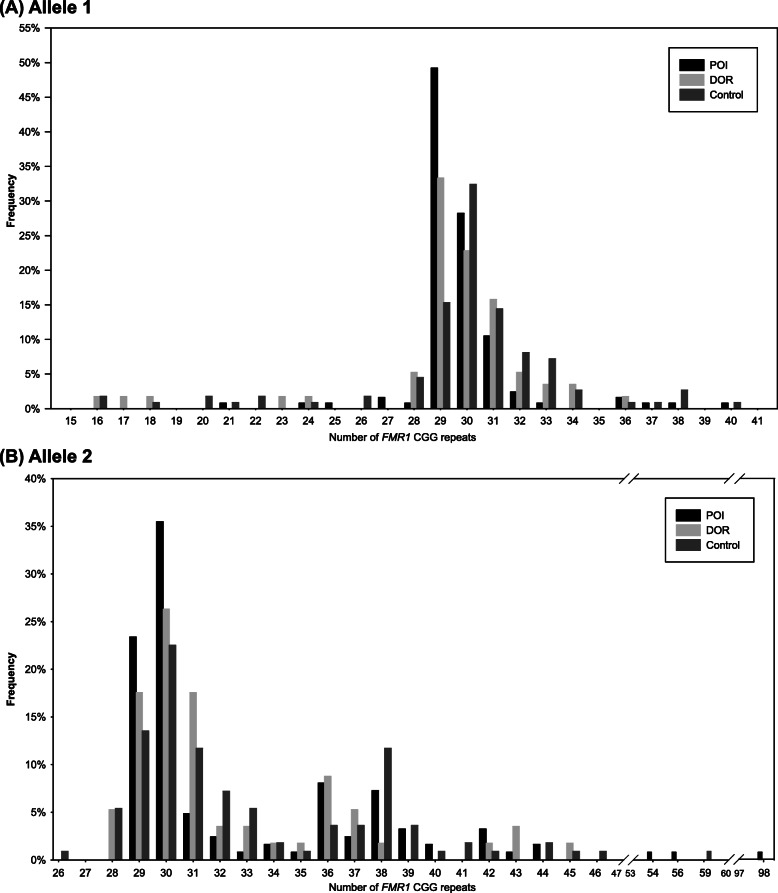
Distribution of *FMR1* CGG repeat numbers in Chinese patients with POI, DOR and controls. **a, b** Data from allele 1 (**a**) and allele 2 (**b**) (*N* = 124, 57 and 111 for patients with POI, DOR and controls, respectively). Abbreviations: *FMR1, the fragile X mental retardation1;* POI primary ovarian insufficiency; DOR diminished ovarian reserve

The *FMR1* CGG repeat distribution in POI, DOR, and control groups is shown in Fig. 1a (allele 1) and Fig. 1b (allele 2). The overall pattern of distribution in different groups is almost similar. The most common CGG repeat of both alleles in all the groups was 29 and 30. The distribution of alleles among groups were compared, and no significant difference was found.

Screening of the *FMR1* gene identified 2 premutation carriers with repeats 31/56, 30/98 in the POI group (1.6%), and 1 with repeats 40/59 in the control group (0.9%). Four intermediate repeat carriers were found: 1 in the POI group (0.8%), 1 in the DOR group (1.8%) and 2 in controls (1.8%). No full mutation subtype was found in all groups. For allele 1, there were no intermediate or premutation carriers. The repeat lengths for allele 2 were in the range of 26–98 in all the participants, and had a secondary peak around 36–39 repeats. Homozygous CGG repeat lengths were common in both the groups (POI group: 50.0%; DOR group: 61.4%; control group: 66.7%), and the commonest repeats were 30/30 and 29/29 repeats in all the groups.

### Distribution of CGG repeats in POI, DOI, and controls

**Fig. 2:**
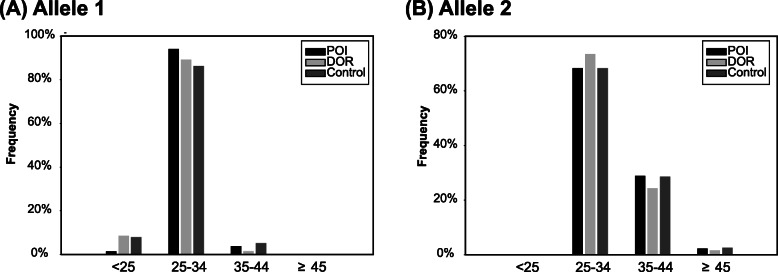
Frequency distribution of CGG repeats in POI, DOR cases and controls in groups < 25, 25–34, 35–44, and ≥ 45. Abbreviations: POI primary ovarian insufficiency; DOR diminished ovarian reserve

The CGG repeats were also divided into groups of five consecutive values (Table 2). The distribution of allele 1 in the POI group was different from that in the control group (*P* < 0.001). No statistically significant differences were found for allele 1 between DOR group vs. controls, for allele 2 between POI group vs. controls, and DOR group vs. controls (*P* > 0.05). The proportion of < 25, 25–34, 35–44 and > 44 group for both the alleles are shown in Fig. 2. Comparing the distribution between the POI vs. controls and DOR vs. controls, the distribution of allele 1 was different between the POI group and the control group (*P* = 0.044), whereas there was no significant difference between the DOR group and controls for allele 1, and for allele 2 between POI or DOR group and controls for allele 2 (*P* > 0.05).

**Table 2:** Detailed distribution by 5 CGG repeat bands up through the premutation in patients with POI, DOR and controls

FMR1 CGG Repeats	N	<25	25–29	30–34	35–39	40–44	45–54 (Intermediate)	55–199 (Premutation)
**Allele1**								
**POI group, N (%)**	**124**	**2 (1.6)**	**65 (52.4)**	**52 (41.9)**	**4 (3.2)**	**1 (0.8)**	**0**	**0**
Age at menopause, yrs., mean (SD)		16.5 (2.1)	28.9 (7.3)	28.7 (6.5)	23.5 (6)	22		
FSH (IU/l), mean (SD)		95.2 (43.3)	79 (36.2)	81.4 (38.5)	67.5 (34.9)	129.3		
AMH (ng/ml), mean (SD)		0	0.1 (0.3)	0 (0.1)	0	0		
**DOR group, N (%)**	**57**	**5 (8.8)**	**22 (38.6)**	**29 (50.9)**	**1 (1.8)**	**0**	**0**	**0**
FSH (IU/l), mean (SD)		17.8 (8.8)	15.5 (4.7)	14.1 (3.9)	16.9			
AMH (ng/ml), mean (SD)		1.0 (0.9)	0.7 (0.6)	0.8 (0.8)	0.9			
**Control group, N (%)**	**111**	**9 (8.1)**	**24 (21.6)**	**72 (64.9)**	**5 (4.5)**	**1 (0.9)**	**0**	**0**
Age at menopause, yrs., mean (SD)		52 (2)	50.5 (3.3)	50.5 (2.9)	52.8 (4)	51.0		
FSH (IU/l), mean (SD)		27.3 (29.5)	26.9 (26.9)	32.5 (32.7)	21.8 (29.6)	52.9		
**Allele2**								
**POI group, N (%)**	**124**	**0**	**29 (23.4)**	**56 (45.2)**	**27 (21.8)**	**9 (7.3)**	**1 (0.8)**	**2 (1.6)**
Age at menopause, yrs., mean (SD)			29.6 (6.7)	27.9 (7.4)	27.6 (7.5)	27.8 (5.5)	30.0	35.5 (2.1)
FSH (IU/l), mean (SD)			87.2 (36.8)	80.8 (39.5)	76.5 (36.2)	70.2 (29.6)	110.5	59.1 (10.3)
AMH (ng/ml), mean (SD)			0.0	0.1 (0.3)	0.0	0.0	0.0	0.1 (0.2)
**DOR group, N (%)**	**57**	**0**	**13 (22.8)**	**30 (52.6)**	**10 (17.5)**	**3 (5.3)**	**1 (1.8)**	**0**
FSH (IU/l), mean (SD)			15.9 (4.7)	14.4 (5.3)	15.3 (3.8)	17.4 (1.5)	11.0	
AMH (ng/ml), mean (SD)			0.5 (0.6)	0.9 (0.9)	0.9 (0.6)	0.4 (0.3)	0.7	
**Control group, N (%)**	**111**	**0**	**22 (19.8)**	**54 (48.6)**	**26 (23.4)**	**6 (5.4)**	**2 (1.8)**	**1 (0.9)**
Age at menopause, yrs., mean (SD)			51.3 (3)	50.5 (3.1)	50.9 (3)	51 (1.9)	46.5 (2.1)	51.0
FSH (IU/l), mean (SD)			23.6 (25.3)	28.8 (30.7)	39.3 (34.5)	15.2 (18.8)	68.9 (32.3)	52.9

Fisher’s exact tests for allele 1: comparison by POI versus control (*P* < 0.001); comparison by DOR versus control (*P* = 0.148). Fisher’s exact tests for allele 2: comparison by POI versus control (*P* = 0.903); comparison by DOR versus control (*P* = 0.884). The menopausal ages, values of FSH and AMH of women with different CGG repeats groups showed no statistically significant difference (*P* > 0.05)

Abbreviation: *POI* Primary ovarian insufficiency, *DOR* Diminished ovarian reserve, *FSH* Follicle-stimulating hormone, *AMH* Anti-mullerian hormone

The association between *FMR1* CGG repeats and the age at menopause was explored in POI women (Table 2). Menopausal ages of women with different CGG repeats groups showed no statistically significant difference (*P* > 0.05). Furthermore, the mean FSH and AMH values did not show any association with different CGG repeats in both the POI and DOR groups (Table 2). Comparison of menopausal age between women having allele 2 with ≤38 repeats (*n* = 100) and > 38 repeats (*n* = 16) revealed no significant difference (28.1 ± 7.3 vs. 30.0 ± 5.1, *P* = 0.321).

## Discussion

In the present study, we compared the distribution of FMR1 CGG repeats among 124 patients with POI, 57 patients with DOR and 111 controls in Chinese population. No statistically significant differences were identified between the DOR group and the control group, whereas the distribution of allele 1 CGG repeat in patients with POI was difference from that in the control group. Frequency of premutation was relatively low in both healthy women and patients with ovarian dysfunction in China: two cases with premutation carriers were identified in the POI group, 1 premutation carrier in the control group, and no carriers were found in the DOR group.

POI occurs in 1% of women and has severe consequences, including infertility and chronic hypoestrogenism that may result in increased cardiovascular risk, impaired bone health, and considerable psychosocial sequelae [32]. A higher prevalence of spontaneous POI has been reported from a very recent meta-analysis, i.e. 3.7% among all women worldwide [33]. Hormone replacement therapy, the principal therapeutic approach for POI, helps alleviate the related symptoms although this does not effectively solve the issues related to fertility.

The main cause of POI is unknown, but genetic factors, autoimmune ovarian damage, iatrogenic and environmental factors are the known causes. Among all the genetic factors implicated for POI, the *FMR1* premutation is regarded as the leading single-gene cause of POI [34]. If *FMR1* screening for the target population could select women with high risk of POI, it has long-term benefits to family and enables them in planning the families with an opportunity to adopt alternate methods. The American College of Obstetricians and Gynecologists (ACOG) and the European Society of Human Reproduction and Embryology (ESHRE) recommend population-based *FMR1* screening for women younger than 40 years of age and presenting with ovarian insufficiency [35, 36]. *FMR1* testing has a dual role in patients with ovarian insufficiency: determining the probable cause of ovarian failure and identifying women at risk of transmitting mutations to their offspring. However, studies have shown wide heterogeneity and inconsistency in the association between *FMR1* CGG repeats and POI susceptibility across different ethnicities [15, 37].

*FMR1* premutation carriers showed an increased risk of POI, especially in a population of European descent [37]. However, this factor may not contribute to the POI susceptibility in the Asian population [37, 38]. Studies of Iranian [39] and Indian [37] populations found no significant relationship between the *FMR1* CGG repeat and POI. Studies from China also showed *FMR1* premutation to be an uncommon explanation for POI [18, 19]. The frequency of premutation carriers among Chinese women with sporadic POI ranges between 0.5 and 0.9% in three previous studies [17–19]; these studies analyzed *FMR1* CGG repeats using PCR and capillary electrophoresis. Our study shows the premutation frequency in the POI group was 1.6% using PCR and microfluidic capillary electrophoresis. A previous study showed that the repeat sizes determined from both methods were largely concordant and, on average, were within one repeat size difference [30]. Then, we speculated that the error rate within the two methods is acceptable. Our results combined with three previous reports in China, shows the premutation frequency in Chinese women with sporadic POI may be 0.87% (6/693). Typically, in healthy women, the premutation carrier frequency has been reported as 1/579–1/1955 in China [40–42], 1/781 in Korea [43]. The permutation frequency in Asian women [41] is much lower than the statistics in white (1/169), African American (1/124), and Hispanic (1/287) healthy women [44]. Although limited by sample size, our study found no statistical difference in the premutation carrier frequency among POI and healthy women. It is possible that the premutation carrier frequency in Chinese POI patients is higher than that in healthy women, but the overall incidence is relatively low in both groups. Further study with a larger sample size is needed to get a definite conclusion.

Studies have reported controversial outcomes, wherein intermediate and normal alleles are associated with POI frequency [13, 14, 19]. Our study, in agreement with others, found the distribution of allele 1 in the POI group was different from healthy women. Another study from China suggested that the CGG repeats in allele 1, and not allele 2, were significantly associated with POI occurrence [17]. The risk of POI occurrence for < 26 and ≥ 29 CGG repeats in allele 1 was higher than that for 26–28 CGG repeats. Gleicher et al. [45] demonstrated that < 26 repeats of both alleles have negative effects on reproduction.

Various studies also explored the association of *FMR1* CGG repeats with DOR. Most of these studies have shown association of DOR with the number of CGG repeats of the *FMR1* gene [5, 9, 21–24, 46], while few other studies including ours suggest that the number of CGG repeats of the *FMR1* gene seems to be independent of DOR [26, 27]. Our study found no difference in the CGG repeats of allele 1 between the DOR and control groups in Chinese women. Pastore et al. [25] found a significant difference in the CGG repeats of allele 1 between the DOR cases and women with a normal reproductive history among the Whites, but not the Asians, since Asian women seem less likely to have an allele with ≤25 CGG repeats than other races [25, 47]. Race variation may be associated with the different results.

The previously reported frequency of 35–44 repeats is 14.5–17% in the DOR group and 3.9% in the controls [5, 23], suggesting that the CGG repeats of 35–44 may be markedly overrepresented in women with DOR. We compared the proportion of 35–44 CGG repeats among groups in this study, and found no difference of the prevalence between the DOR group and control. In the control group, which included women with normal menopausal age, the frequency of 35–44 CGG repeats is 28.8%, which is much higher than in previous reports. The difference might be attributable to the presence of the secondary modal peak seen in the Asian population. Women in the present study had a primary modal peak at 29 to 31 repeats and a secondary modal peak at 36 to 38 repeats of allele 2. Reports showed other Asians such as Japanese [12, 48] and Indonesians [41] also have a secondary peak, which was not identified in studies of western populations [12]. The presence of a secondary modal peak may be related to varied outcomes across different races.

The underlying mechanism behind CGG repeats regulating *FMR1* gene expression in the ovary, and thereby affecting ovarian function remains unknown. It is unclear whether the reduced ovarian reserve represents a pathological condition resulting from abnormally accelerated atresia in a normal antral follicular pool or an abnormally small initial pool of oocytes [49]. A recent study showed that various CGG expansions of an *FMR1* allele may lead to changes in RNA level and ratios of distinct RNA isoforms, which could regulate the translation and/or cellular localization of fragile X mental retardation protein (FMRP), affecting the expression of steroidogenic enzymes and hormonal receptors, that result in ovarian dysfunction [50]. In addition, Dioguardi et al. found that the permutation transcript contributes to the mitochondrial and ovarian abnormalities in permutation mouse models [51]. Studies showed that ovaries from Fmr1 knockout mice show increased mTOR protein [52, 53], and the YAC mice with premutation CGG repeat show reduced phosphorylated mTOR levels [54]. Then, both underexpression and overexpression of mTOR can result in ovarian dysfunction [55]. A recent study showed a potential relationship between the regulation of FMR1/FMRP expression and the AKT/mTOR signaling pathway in a human proliferating granulosa cell model system [56]. The above experiments suggest the mTOR pathway as a potential therapeutic target. However, the result of CGG repeats affecting the ovarian ageing process in various ethnicities is inconsistent, and the mechanism of heterogeneity in varied ethnicity is unclear. Further functional studies are needed to explain the inconsistent results across different ethnicities and susceptibility to ovarian insufficiency.

The primary strength of this study was the identification of women with a well-defined phenotype, independent of any potential risk factors for analysis of *FMR1* CGG repeats. Second, as the association *FMR1* gene and DOR has not been evaluated, by enrolling both POI and DOR patients in this study, we could have a comparison between these two groups in one Center. The primary limitation of the study however is the relatively small sample size.

## Conclusions

No difference of *FMR1* alleles in the premutation ranges were found between POI or DOR and healthy women and the frequency of premutation was relatively low in Chinese women. The distribution of allele 1 CGG repeat in patients with POI showed some different from that in healthy women. Although *FMR1* testing has been recommended in the evaluation of the etiology of ovarian insufficiency in western countries, screening for the same among Chinese women is not warranted as the low frequency of occurrence. Further investigations with larger sample sizes is necessary to study the incidence of *FMR1* expansions in all forms of ovarian insufficiency to confirm the results of this pilot study.

## Data Availability

The datasets used and/or analysed during the current study are available from the corresponding author on reasonable request.
